# The effect of sodium bicarbonate and validation of beckman coulter AU680 analyzers for measuring total carbon dioxide (TCO_2_) concentrations in horse serum

**DOI:** 10.1002/vms3.82

**Published:** 2017-10-25

**Authors:** Levent Dirikolu, Pamela Waller, Mona Landry Waguespack, Frank Michael Andrews, Michael Layne Keowen, Stephen David Gaunt

**Affiliations:** ^1^ Equine Medication Surveillance Laboratory Department of Comparative Biomedical Sciences School of Veterinary Medicine Louisiana State University Skip Bertman Drive Baton Rouge Louisiana 70803; ^2^ Equine Health Studies Program Veterinary Clinical Sciences Louisiana State University 1843 Veterinary Teaching Hospital Baton Rouge Louisiana 70803; ^3^ Louisiana Animal Disease Diagnostic Laboratory Department of Pathobiological Sciences School of Veterinary Medicine Louisiana State University Skip Bertman Drive Baton Rouge Louisiana 70803

**Keywords:** Horses, milkshake, sodium bicarbonate, TCO_2_

## Abstract

This study evaluated the usage of Beckman Coulter AU680 analyzers for measurement of TCO_2_ in horse serum, and the effect of sodium bicarbonate administrations on serum TCO_2_ levels in resting horses. Treatment of horses with sodium bicarbonate did not result in any adverse events. Mean TCO_2_ concentration was significantly higher from 1 to 8 h in the sodium bicarbonate‐treated horses compared to the untreated controls. Within an hour, administration of sodium bicarbonate increased the TCO_2_ level from 31.5 ± −2.5 (SD) to 34.0 ± 2.65 (SD) mmol/L and at 2–8 h post‐administration, the TCO_2_ level was above the 36 mmol/L cut‐off level. In all quality control analysis of Australian standard by Beckman Coulter AU680 analyzer, the instrument slightly over estimated the TCO_2_ level but the values were in close agreement with mean TCO_2_ level being 38.03 with ± 0.87 mmol/L (SD). Expanded uncertainty was calculated using different levels of confidence interval. Based on 99.5% confidence interval using 0.805% expanded uncertainty using mean measured concentration of 38.05 mmol/L, it was estimated that any race samples TCO_2_ level higher than 38.5 mmol/L will be indicative of sodium bicarbonate administration using Beckman Coulter AU680 analyzer in Louisiana.

## Introduction

Sodium bicarbonate given by nasogastric tube has been used by some trainers as the key ingredient in a ‘milkshake’ (Lloyd *et al*. 1993; Frey *et al*. 1995; Manohar *et al*. 2004). It has been suggested that such treatment given 3–5 h prior to racing may enhance a horse's racing performance by increasing the blood buffering capacity and enhancing lactate clearance from skeletal muscle, thereby delaying the onset of fatigue (Sutton *et al*. 2014). During prolonged exercise, lactic acid builds up in the muscle of horses, lowering the blood pH (Beard & Hinchcliff 2002). A mixture of alkalinizing salts, usually sodium bicarbonate, is given by some trainers in a process called ‘milkshaking’ to increase the blood buffering capacity in order to remove lactate from the skeletal muscle, delaying fatigue (Lloyd & Rose 1995; Waller *et al*. 2010).

Horsemen who train performance horses, and in particular racehorses, assume that administering a large dose of sodium bicarbonate (about 500 g in a slurry of electrolytes, sugar and water) can boost performance (Lloyd *et al*. 1992). Although studies have shown performance enhancement in humans, milkshaking's effectiveness has shown mixed results in horses (Wilkes *et al*. 1983; Harkins & Kamerling 1992). Traditionally administered via nasogastric tube prior to a race, sodium bicarbonate (milkshakes) might be effective in neutralizing acid in muscle cells by raising the pH of the blood and, therefore, delaying the onset of fatigue in working muscle, which might permit the horse to go longer at peak rate of endurance or speed. During racing, controlling the pH of the blood is crucial in counteracting the buildup of lactic acid and other toxins that are produced from exercising muscle. When muscle contracts for extended periods of time at fairly high speed, the interior of the muscle cell becomes highly acidic, resulting in the progressive failure of energy‐producing pathways and leading to fatigue. Introducing bicarbonate into the system does not change the pH of the muscle cell, only the blood. Bicarbonate, called a base, is a natural component of blood and it is especially effective at neutralizing lactic acid produced during maximal exercise. When sodium bicarbonate present in sufficient or excessive amounts in the blood stream, such as during times of intensive exercise, acid in the muscle pulled out of the cell into the blood stream. There it reacts with sodium bicarbonate to form a compound called carbonic acid, which is a weak acid (Lloyd & Rose 1995). It is believed that sodium bicarbonate administration increases the blood buffering capacity in order to remove lactate from skeletal muscle, delaying fatigue (Heffron *et al*. 2014). Another concern regarding the use of sodium bicarbonate (milkshakes) is hiding or masking the use of other drugs. It is theorized that the administration of alkaline substances such as sodium bicarbonate can affect the excretion of some drugs (Lloyd *et al*. 1992). Drugs that are of a basic nature might be excreted in lower concentrations and for a longer duration, while those that are more acidic could be excreted more rapidly. This masking effect seems to be most effective for drugs such as lidocaine, procaine and cocaine.

Horses consuming pasture grasses obtain a natural, endogenous level of bicarbonate in the plasma in the range of 26–31 mmol/L (Gill 2004). Performance horses are fed commercial forage and grain preparations, which contain higher amounts of bicarbonate. Therefore, the level of TCO_2_ in their plasma increases to a range of about 27–32 mmol/L but rarely exceeds 33 mmol/L (Gill 2004). Attempts to manipulate racing performance using sodium bicarbonate were identified in the early 1990s (Auer *et al*. 1993). In response, Louisiana State Racing Commission (LSRC) set the limit for total carbon dioxide (TCO_2_) concentrations in plasma/serum at 36.0 milliequivalents per litre (mmol/L). Total CO_2_ (TCO_2_) is the combination of all forms of carbon dioxide in plasma that are in equilibrium in blood (Sutton *et al*. 2014). As such, the plasma or serum concentration of TCO_2_ provides a clinically useful screening test for the presence of metabolic acid base disturbances, including the prerace administration of alkalinizing agents to racehorses. According to the Louisiana Horse Racing Guidelines, the state veterinarian may draw blood samples from a horse for obtaining a TCO_2_ concentration level. Blood samples for TCO_2_ may be drawn prior to, or after, the race. Samples drawn after the race shall not be drawn earlier than 90 min following official post time. Samples drawn pre‐race shall be drawn prior to the official post time. The pre‐ or post‐race TCO_2_ level in the blood shall not exceed 36.0 (mmol/L). Our laboratory uses the Beckman Coulter AU680 as the instrument for screening and confirmatory quantitation as it is considered suitable for analysis. The purpose of this study was to evaluate the usage of Beckman Coulter AU680 analyzers for measurement of TCO_2_ in horse serum, and to determine the effect of sodium bicarbonate administrations on serum TCO_2_ levels in horses. To our knowledge, the effect of administration of sodium bicarbonate on TCO_2_ levels have not been investigated in horses, although there have been several studies conducted to determine the storage and handling, seasonal temperature variation, track location, thoroughbred status, pre‐ or post‐race, furosemide administration status, dietary, environmental and handling practices and health concerns on TCO_2_ levels in racehorses (Heffron *et al*. 2014; Sutton *et al*. 2014; Waller *et al*. 2010; Cohen *et al*., 2006).

## Materials and methods

### Instrumentation and quality control

A Beckman Coulter AU680 analyzer (Beckman Coulter, Indianapolis, IN) instrument was calibrated daily before samples were analyzed. Beckman Calibrators were obtained from Beckman Instruments and stored at 4°C prior to analysis at 20 and 40 mmol/L. Controls were from Biorad‐Liquid Unassayed Multiqual Levels at 14.86, 18.46, 28.39 mmol/L (Clinical Diagnostics, Hercules, CA), and 36.01 (±0.17) mmol/L control was from Australian Scientific Enterprise (Australian Scientific Enterprise P/L, Hornsby NSW, Australia) stored at 4°C prior to analysis. Four replicates of four quality control samples in addition to calibration standards were analyzed before and after each batch daily for total of five days. Linearity of the test is stated as 5‐50 mmol/L (0, 5, 10, 20, 30, 40, 50).

This study used Association of Official Racing Chemist (AORC) protocol for cross validating TCO_2_ measuring instrument to validate Beckman Coulter AU680 analyzer. Both within‐run and inter‐day reproducibility were demonstrated in this section of the validation for the Beckman Coulter AU680 using four quality control samples at 14.86, 18.46, 28.39 and 36.01 mmol/L in addition to calibration standards run in quadruplicates daily for a total of 5 days. The quantitative method was validated by examining the measurement of consistency of results (within‐run and between‐run). The within‐run precision was calculated from similar responses of eight repeats of four quality control samples while between‐run precision was determined by comparing the calculated response to actual TCO_2_ concentrations in quality control samples over five consecutive daily runs (total of 40 runs). Statistical analysis and calculation of expanded uncertainty were done using Microsoft excel 2016 program. For all comparisons, hypothesis testing was two‐tailed, and *P* < 0.05 was considered significant. Expanded uncertainty was estimated according to the National Measurement Institute (NMIA) standard procedures. Standard uncertainty was estimated as described in ISO Guide to the Expression of Uncertainty in Measurement. TCO_2_ concentration is reported with an expanded uncertainty (*U*). The expanded uncertainty is calculated as *U* = *k*xu, where *k* is the coverage factor and *u* is combined standard uncertainty. The expanded uncertainty was calculated at different confidence intervals (95, 97.5, 99 and 99.5%).

### Horses and sample collections

Four healthy Thoroughbred horses ages 4–11 years old were selected from the Louisiana State University Equine Health Study Program herd and studied in a 2 × 2 cross over design. Horses were fed twice a day. The animals were vaccinated annually and dewormed quarterly. A routine clinical examination was performed before each experiment to assure that the animals were healthy and sound. Horses were housed in stalls throughout the study and fed grain (Purina^®^ Strategy, Purina Mills, Grey Summit, MO) and mixed grass hay twice daily (around 10 am and 2.30 pm). The horses were fasted for 12 h before and at least 2 h after sodium bicarbonate administration. For phase 1, two horses were randomly selected to receive the sodium bicarbonate and the other two horses were untreated controls. After a week of wash‐out period, the horses were crossed over. This study was approved by the Louisiana State University Institutional Animal Care and Use Committee.

Two blood samples were collected in separator tubes (BD Vacutainer^®^ tubes, BD Franklin Lakes, NJ) without anticoagulant before sodium bicarbonate administration, then every hour for 8 h, and then 24, 48, 72 and 96 h after treatment. Blood samples were left at room temperature to allow clot formation for 50 min, then centrifuged at 1300*g* for 15 min, then stored at 4°C overnight. Serum (TCO_2_) was measured in quadruplicate using the Beckman Coulter AU680 analyzer. ‘Milkshake’ (Sodium bicarbonate, 500*g*. in 2 L tap water, Arm & Hammer Pure Baking Soda) was administered through a nasogastric tube. The time between blood collection and analysis was 24–72 h. This time frame is in agreement with previously published data on TCO_2_ stability (Tinkler *et al*. 2012). Basic handling procedures for samples were as follows: (1) Fill tubes to the stated draw volume to ensure the proper blood‐to‐additive ratio. Allow the tubes to fill until the vacuum is exhausted and blood flow ceases; (2) Mix all gel barrier and additive tubes by gentle inversion 5–10 times immediately after the draw. This assists in the clotting process. This also assures homogenous mixing of the additives with the blood in all types of additive tubes; (3) Serum separator tubes should clot for a minimum of 30 min in a vertical position prior to centrifugation. Short clotting times can result in fibrin formation, which may interfere with complete gel barrier formation. Gel barrier is temperature related; flow may be impeded if chilled before or during centrifugation; (4) Blood Sample Centrifugation: It is recommended that serum be physically separated from contact with cells as soon as possible, with a maximum time limit of 1 h from the time of collection; (5) TCO_2_ tubes should never be frozen or have top removed for any reason and tubes should not be placed directly on ice packs.

## Results

**Figure 1:**
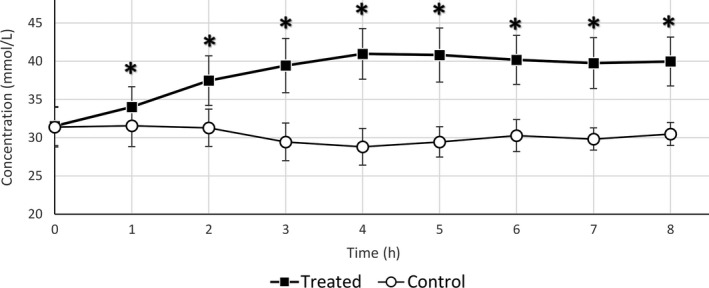
Mean ± SD TCO_2_ concentrations at 0–8 h in control and sodium bicarbonate treated animals. Stars signify significant difference *P* < 0.05 compared to same time point.

**Figure 2:**
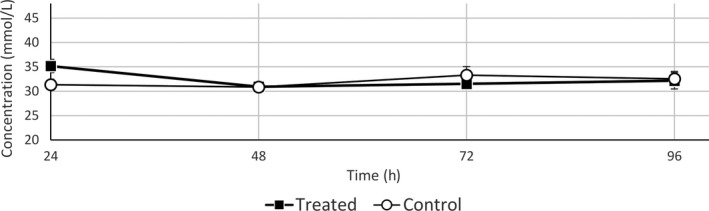
Mean ± SD TCO_2_ concentrations at 24, 48, 72 and 96 h in control and treated animals.

Treatment with sodium bicarbonate (milkshake) did not result in any adverse events. When data were pooled, the mean TCO_2_ concentration before treatment (time = 0) was not significantly different, so horses had similar starting values in phase 1 and phase 2 studies. Mean TCO_2_ concentration was significantly higher from 1 to 8 h in the sodium bicarbonate‐treated horses compared to the untreated controls (Fig. 1). Within an hour, administration of sodium bicarbonate increased the TCO_2_ level from 31.5 ±  2.5 (SD) to 34.0 ± 2.65 (SD) mmol/L and at 2–8 h post‐administration, the TCO_2_ level was above the 36 mmol/L cut‐off level. The mean TCO_2_ concentrations did not differ significantly between the sodium bicarbonate‐treated and control horses at times 24, 48, 72 and 96 h (Fig. 2).

The mean within‐run and between‐run accuracies for quality control samples at 14.86, 18.46 and 28.39 mmol/L were 96.3% (±5.70 SD), 100.9% (±2.40 SD) and 97.2 (±5.4 SD), respectively (Table 1). The mean within‐run and between‐run coefficient of variations (CV) for quality control samples at 14.86, 18.46 and 28.39 mmol/L were 5.92, 3.63 and 5.5%, respectively (Table 1). The Australian quality control standard is recommended by AORC for validating instruments for measuring TCO_2_ in racehorses. According to the manufacturer, the TCO_2_ level provided to our laboratory had 36.01 (±0.17) mmol/L TCO_2_ level. In order to have the highest margin of the error in our data, it was assumed that the level in this quality control was 35.84 (36.01–0.17) mmol/L. In all quality control analysis of Australian standard by Beckman Coulter AU680 analyzer, the instrument slightly over estimated the TCO_2_ level but the values were in close agreement with mean TCO_2_ level being 38.03 with ±0.87 mmol/L (SD) (Table 2). The mean within‐run and between‐run accuracies for quality control samples at 35.84 mmol/L were 106.0% (±2.42 SD) and 106.2% (±1.61 SD), respectively (Table 2). The mean within‐run and between‐run coefficient of variations (CV) for quality control samples at 35.84 mmol/L were 2.28 and 1.82%, respectively (Table 2). Expanded uncertainty was calculated using different levels of confidence interval. The usage of 99.5% confidence interval with 0.805% expanded uncertainty using mean measured concentration of 38.05 mmol/L provided 38.41 mmol/L (~38.5 mmol/L) (Table 2). Based on this data, any race samples TCO_2_ level higher than 38.5 mmol/L will be indicative of sodium bicarbonate administration in racehorses using Beckman Coulter AU680 analyzer in Louisiana.

**Table 1:** The mean within‐run and between‐run accuracies and CVs for quality control samples at 14.86, 18.46 and 28.39 mmol/L

Theoretical concentration	Measured concentration (mean ± SD)	Accuracy (%) (mean ± SD)	Coefficient of variation (%)
Within‐run (*n* = 8)			
14.86	14.30 ± 0.85	96.3 ± 5.70	5.92
18.46	18.63 ± 0.44	100.9 ± 2.40	3.63
28.39	27.59 ± 1.53	97.17 ± 5.39	5.55
Mean		98.12	5
Between‐run (*n* = 40)			
14.86	14.62 ± 0.63	98.4 ± 4.21	4.28
18.46	18.46 ± 0.53	99 ± 2.85	2.85
28.39	28.22 ± 1.04	97.41 ± 3.67	3.70
Mean		98.3	3.61

**Table 2:** The mean within‐run and between‐run accuracies and CVs for Australian quality control sample at 36.01 (±0.17) mmol/L

Theoretical Concentration	Measured concentration (mean ± SD)	Accuracy (%) (mean ± SD)	Coefficient of variation (%)
Within‐run (*n* = 8)			
35.84	38.03 ± 0.87	106.0 ± 2.42	2.28
Between‐run (*n* = 40)			
35.84	38.05 ± 0.61	106.2 ± 1.61	1.82

Standard uncertainty (Standard error)	Confidence interval (%)	Expanded uncertainty (%)
0.2705	95	0.5471
	97.50	0.6306
	99	0.7324
	99.50	0.8048

In order to have the highest margin of safety, it was assumed that the level in this quality control sample was 35.84 (36.01–0.17) mmol/L. Expanded uncertainty was also calculated based on different levels of confidence interval.

## Discussion and conclusion

Total CO_2_ (TCO_2_) is the combination of all forms of carbon dioxide in plasma that are in equilibrium in blood. As such, the plasma or serum concentration of TCO_2_ provides a clinically useful screening test for the presence of metabolic acid base disturbances, including the prerace administration of alkalinizing agents to racehorses. Horse racing authorities impose a limit on the concentration of plasma TCO_2_, typically 36 mmol with action taken above 37 mmol, as measured by an electrochemical gas analyzer (Sutton *et al*. 2014).

To the best of our knowledge, this is the first study conducted to validate Beckman Coulter AU680 analyzer to measure TCO_2_ in racehorse's samples, and this is the first published data investigating the effect of sodium bicarbonate administration on total serum TCO_2_ levels in hourly bases for the first 8 hr post‐administration. In this study, it was shown that treatment with sodium bicarbonate did not result in any adverse events and administration of sodium bicarbonate significantly increases TCO_2_ level within an hr and this effect last up to 8 h or even longer in resting horses. Unfortunately, only first 8 h samples were collected after sodium bicarbonate administration, so it remains unknown for how long the administration of sodium bicarbonate may increase the TCO_2_ level above the cut‐off limit of 36 mmol/L before reaching the baseline level within 24 h. It is important to emphasize that only four adult horses were used in this study to investigate the effect of sodium bicarbonate on serum TCO_2_ levels in horses in resting horses. Therefore, further studies are warranted using larger number of exercised animals to determine the effect and the duration of the effect of sodium bicarbonate on total serum TCO_2_ levels especially after 8 h post‐administration on hourly basis for up to 24 h in racehorses. The TCO_2_ level returns back to baseline level within 24 h after sodium bicarbonate administration. If it is assumed that increased TCO_2_ level in racehorses has performance enhancing effect, administration of sodium bicarbonate increases the racing performance within an hour that last at least up to 8 h based on data obtained in this study.

In Louisiana, the state veterinarian may draw blood samples from a horse for obtaining a TCO_2_ (total dissolved carbon dioxide) concentration level. Blood samples for TCO_2_ may be drawn prior to, or after, the race. Samples drawn after the race shall not be drawn earlier than 90 min following official post time. Samples drawn pre‐race shall be drawn prior to the official post time. The pre‐ or post‐race TCO_2_ level in the blood shall not exceed 36.0 mmol/L. In our laboratory, the Beckman Coulter AU680 analyzer is used for measuring TCO_2_ level in racehorses in order control usage of sodium bicarbonate in racehorses. In all quality control analysis of Australian standard by Beckman Coulter AU680 analyzer, the instrument slightly over estimated the TCO_2_ level but the values were in close agreement with mean TCO_2_ level being 38.03 with ±0.87 mmol/L (SD). Based on 99.5% confidence interval using 0.805% expanded uncertainty using mean measured concentration of 38.05 mmol/L, it was estimated that any race samples TCO_2_ level higher than 38.5 mmol/L will be indicative of sodium bicarbonate administration in racehorses using Beckman Coulter AU680 analyzer in Louisiana. A complication to determining the baseline TCO_2_ level in the horse is the administration of furosemide, which is used in horse racing to treat exercise‐induced pulmonary haemorrhaging, a common malady in racehorses. Furosemide has been known to increase the plasma TCO_2_ concentrations in horses. A side effect of furosemide administration is increased urine output. Because of this increase, the horse's strong ion concentrations are affected. This change in strong ion concentrations could affect the retention of bicarbonate in the bloodstream increasing the concentration of TCO_2_ (Freestone *et al*. 1988, 1989; Carlson & Jones 1999). It is important to emphasize that the potent diuretic agent furosemide (Lasix^®^) is an alkalizing agent that causes about a 2 mmol/L increase in TCO_2_. The racing authorities should take the effect of furosemide on TCO_2_ level into consideration when evaluating whether or not the horse was given sodium bicarbonate as a performance enhancing medication (Heffron *et al*. 2014; Kline *et al*. 2006).

## Source of funding

The source of funding was from contract obtained from Louisiana Sate Racing Commission, New Orleans, LA, USA.

## Conflicts of interest

The authors declare no financial or non‐financial interest in the subject matter or materials discussed in this manuscript.

## Contribution

The authors thank Louisiana State University Equine Health Study personnel for helping with sample collection and maintaining horses for this study.

## Ethics statement

All applicable international, national, and/or institutional guidelines for the care and use of animals were followed. All procedures performed in studies involving animals were in accordance with the ethical standards of the institution or practice at which the studies were conducted.
